# Microbicide excipients can greatly increase susceptibility to genital herpes transmission in the mouse

**DOI:** 10.1186/1471-2334-10-331

**Published:** 2010-11-18

**Authors:** Thomas R Moench, Russell J Mumper, Timothy E Hoen, Mianmian Sun, Richard A Cone

**Affiliations:** 1 ReProtect, Inc, Baltimore, MD, 21286 USA; 2 Division of Molecular Pharmaceutics and the Center for Nanotechnology in Drug Delivery, UNC Eshelman School of Pharmacy, University of North Carolina at Chapel Hill, Chapel Hill, NC, 27599 USA; 3 Johns Hopkins University, Baltimore, MD, 21218 USA

## Abstract

**Background:**

Several active ingredients proposed as vaginal microbicides have been shown paradoxically to *increase *susceptibility to infection in mouse genital herpes (HSV-2) vaginal susceptibility models and in clinical trials. In addition, "inactive ingredients" (or excipients) used in topical products to formulate and deliver the active ingredient might also cause epithelial toxicities that increase viral susceptibility. However, excipients have not previously been tested in susceptibility models.

**Methods:**

Excipients commonly used in topical products were formulated in a non-toxic vehicle (the "HEC universal placebo"), or other formulations as specified. Twelve hours after exposure to the excipient or a control treatment, mice were challenged with a vaginal dose of HSV-2, and three days later were assessed for infection by vaginal lavage culture to assess susceptibility.

**Results:**

The following excipients markedly increased susceptibility to HSV-2 after a single exposure: 5% glycerol monolaurate (GML) formulated in K-Y^® ^Warming Jelly, 5% GML as a colloidal suspension in phosphate buffered saline, K-Y Warming Jelly alone, and both of its humectant/solvent ingredients (neat propylene glycol and neat PEG-8). For excipients formulated in the HEC vehicle, 30% glycerin significantly increased susceptibility, and a trend toward increased HSV-2 susceptibility was observed after 10% glycerin, and 0.1% disodium EDTA, but not after 0.0186% disodium EDTA. The following excipients did not increase susceptibility: 10% propylene glycol, 0.18%, methylparaben plus 0.02% propylparaben, and 1% benzyl alcohol.

**Conclusions:**

As reported with other surfactants, the surfactant/emulsifier GML markedly increased susceptibility to HSV-2. Glycerin at 30% significantly increased susceptibility, and, undiluted propylene glycol and PEG-8 greatly increased susceptibility.

## Background

Topically administered microbicides are placed in the vagina or rectum where they are intended to prevent infections by blocking pathogens before or soon after entry, advantageously reducing systemic exposures inherent with oral dosing. However, topical application places microbicide formulations at sites where they risk altering epithelial barrier function. Indeed several microbicide Phase III trials have reported strong trends toward or significant *increases *in HIV infection in the active microbicide arms [1-4].

The intact cervicovaginal epithelium provides a substantial barrier against HIV infection. The rate of heterosexual male to female HIV transmission reported in a comprehensive review averaged about 1 in 1250 acts; even during the highly infectious stage immediately after HIV acquisition in the male partner the rate averaged less than 1 in 100 acts [5]. In both the SIV macaque and the FIV macaque models, 10,000 times more virus was required for infection via vaginal challenge than via parenteral challenge [6,7]. It follows that a topical microbicide that compromises this normally potent cervicovaginal barrier could substantially increase susceptibility to HIV or other pathogens.

Traditional safety assessments of topical agents have relied on gross, microscopic, or colposcopic examinations of exposed tissue in animal and clinical studies to detect and quantify tissue damage and inflammation. Recently, more sensitive assessments of microbicide-induced inflammation have been described employing measurement of soluble cytokines [8], and by immunohistochemical staining of tissue inflammatory markers [9]. The slug mucosal irritation assay has been developed as a rapid and quantitative screening test to assess the irritative potential of agents applied to mucosal surfaces [10,11], and to assess diverse topical products and their ingredients. While valuable, both the traditional and these more recent methods assess *surrogate *markers rather than directly testing for toxicities that increase susceptibility to pathogen transmission, arguably the most dangerous of potential microbicide toxicities.

We developed a mouse model that directly determines whether microbicides cause, by any mechanism, toxicity that increases the susceptibility of the vagina to the viral STI pathogen HSV-2 [12]. We found that a single exposures to the candidate microbicides nonoxynol-9 and C31G caused a marked increase in susceptibility to HSV-2, concordant with the increased susceptibility to HIV with nonoxynol-9 observed in a Phase III clinical microbicide trial [2], and a trend toward increased transmission in a Phase III trial of C31G [3]. A similar model has been reported, extending the evaluation to include repeated test agent exposures, and likewise demonstrated increased susceptibility to HSV-2 after multiple nonoxynol-9 exposures [13]. Exposure to cellulose sulfate was recently reported to increase susceptibility to HSV-2 in this multiple-exposure model [14], concordant with the results of the per protocol analysis of Phase III trial where cellulose sulfate was associated with a significantly increased transmission of HIV [4]. These results support the utility of these mouse HSV-2 susceptibility models for predicting microbicide-induced toxicities that increase HIV susceptibility in women.

Certain active pharmaceutical ingredients (APIs) such as nonoxynol-9 have been shown to damage the epithelial barrier in traditional animal and clinical studies [15], and nearly all safety testing has focused exclusively on detecting toxicities of microbicide APIs. Indeed, a commonly used study design has been to compare the active product to a vehicle control (an identical formulation minus the API), based on the belief that excipients are nontoxic. However, this standard 'vehicle control' study design inherently precludes detecting toxic effects of the vehicle that might increase susceptibility to infection. Although excipients are often called "inactive ingredients" and are widely considered to be benign, these ingredients do have activities and toxicities, and none of the excipients employed in microbicide development and widely used in sexual lubricants or other vaginal products have been tested in a manner that would disclose whether or not they alter barrier functions or otherwise increase HIV/STI susceptibility at mucosal surfaces.

## Methods

### Excipients and other materials

Propylparaben, methylparaben, sorbic acid, benzyl alcohol, EDTA disodium dihydrate, glycerin, propylene glycol, and PEG-8 (polyethylene glycol, monomer number = 8, formula weight = 400 Daltons) were U.S.P. or N.F. grades from Spectrum Chemicals & Laboratory Products, Gardena, CA. Hydroxyethylcellulose (HEC, NATROSOL^®^, 250HX PHARM), was from Hercules Incorporated, Wilmington, DE. Bartels^® ^Tissue Culture Refeeding Medium was from Trinity Biotech, St. Louis, MO. Sodium chloride was from Fisher Scientific, Fair Lawn, NJ. Phosphate buffered saline (PBS, Dulbecco's phosphate buffered saline) was from Sigma Aldrich, St. Louis, MO. K-Y^® ^Warming Jelly/Gelle was from Johnson & Johnson Consumer Companies Inc, Skillman NJ. Glycerol monolaurate (GML, Monomuls 90-L 12) was from Cognis Corporation, Cincinnati, OH. Medroxyprogesterone acetate injectable suspension, USP was from Sicor Pharmaceuticals, Inc. Irvine, CA.

### Virus

HSV-2 Strain G (ATCC #VR-734, 2.8 × 10^7 ^TCID_50 _per mL, was from American Type Culture Collection, Manassas, VA), and was aliquoted and stored at- 85°C until thawed for use. When thawed aliquots of this stock were diluted 100-fold with Bartels medium, a 10 μL vaginal dose infected ~50% of the control animals (1 intravaginal infectious dose_50_, or ID_50_).

### Cell lines

Human foreskin fibroblasts in 96-well plates were from Diagnostic Hybrids Inc. Athens, OH.

### Animals

Female Hsd:NSA ™(CF-1^®^) out bred mice were 6-8 weeks old when obtained from Harlan Laboratories, Indianapolis, IN, and were 7-12 weeks old at the time of the studies. These mice were easily handled and no anesthesia was used to deliver the test agents vaginally.

### Preparation of formulations

The HEC "universal" placebo gel formulation (HEC placebo) [16,17], currently being used extensively in microbicide trials [18], was used as a vehicle to which compatible excipients were added for tests in the susceptibility model. The HEC placebo gel formulation contains 2.7% HEC as the gel-forming polymer, 0.85% sodium chloride for tonicity adjustment, 0.1% sorbic acid as a preservative, and sodium hydroxide q.s. to pH 4.4. All percentages were % w/w.

Sorbic acid was omitted in gels formulated for testing of alternative preservatives. GML was formulated as a 5% (w/w) solution in K-Y Warming Jelly (KYWJ), as described in publications assessing GML as a vaginal microbicide [19,20]. To study GML (an agent with limited aqueous solubility in water) in a formulation lacking the extreme hyperosmolality of KYWJ, a colloidal suspension of 5% (w/v) GML in PBS was prepared by stirring GML in PBS with a magnetic stirrer at 50°C for one hour, and maintaining it at 37°C until vaginal administration within 3 hours of preparation, with restirring immediately before administration. Microscopic examination showed this preparation to be a colloidal suspension of ~25 micron diameter GML spherical globules, which remained stable in size for at least 6 hours at 37°C. Concentrations of other excipients were chosen to span substantial portions of the range of concentrations used in vaginal or other topical formulations [21]. The humectant/solvents of KYWJ were tested neat to determine their individual effects at extremely high osmolalities similar to that of the parent KYWJ formulation.

### Osmolality measurements

Osmolality was measured with a vapor pressure osmometer (VAPRO™ Model 5220 Wescor Inc., Logan, UT). For agents or formulations with osmolality above the range of the instrument (GML in KYWJ, KYWJ, PEG-8, and propylene glycol), the osmolality was estimated by linear extrapolation of a 1:3 (v/v) dilution of the product with distilled water.

### Dose-response relationship

To obtain the dose-response relationship, thawed aliquots of HSV-2 stock were diluted sequentially to determine the fraction of mice infected as a function of viral intravaginal dose to cover the range from 0.1 ID_50 _to 10 ID_50_.

### HSV-2 susceptibility model

The procedures were as previously described in detail [12]. Briefly, female 7-12 week old CF-1 mice were injected subcutaneously with 2.5 mg of medroxyprogesterone acetate, and one week later received 20 μL of test agent or PBS control intravaginally with a fire-polished positive displacement capillary pipette (Wiretrol^®^, Drummond Scientific, Broomall, PA). Twelve hours later, mice were challenged with a 10 μL inoculum containing 1 or 0.1 ID_50 _of HSV-2 in Bartels medium as specified below. Mice were assessed for infection three days after inoculation by culturing a PBS vaginal lavage on human foreskin fibroblasts. Prior studies have demonstrated that viral isolation gives very similar but slightly more sensitive detection of infection than assessing HSV-2 disease (visible lesions) [22].

### Statistical analysis

**Figure 1:**
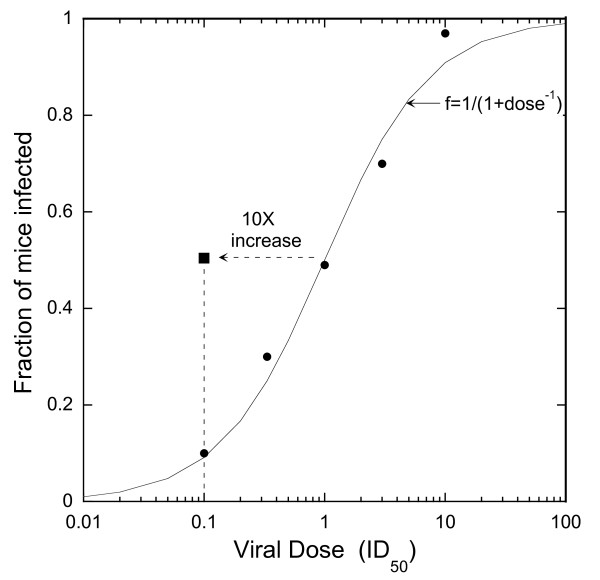
**The filled circles are the averages for groups of 60 mice for each viral dose**. The curve is the best fit of the Michaelis-Menten relationship, f = 1/(1 + dose^-1^), for the dose response data. The black square shows that 50% of animals treated with GML in KYWJ became infected with a viral dose of 0.1 ID_50_, and the dashed arrow indicates that this treatment caused a 10-fold increase in susceptibility (see Table 2).

Data were analyzed by two-sided Fisher's exact test comparing the number of animals infected after exposure to the test agent vs. the number of control animals infected after exposure to PBS using equal size groups of test and control animals. For agents that showed a significant increase in susceptibility, the increase in susceptibility was calculated as the effective infectious dose read from the dose response curve (Figure 1) divided by the infectious dose of the same inoculum in the control animals. Sample size calculations were by standard methods, with an incorporated continuity correction [23], using proportions calculated from the dose-response curve.

## Results

### Dose response relationship

The dose-response relationship between viral dose and infectivity in progestin treated mice is shown in Figure 1. A 100-fold dilution of the viral stock yielded 1 ID_50 _in the 10 μL inoculum volume. When displayed on a semi-log plot, the results show a sigmoid dose-response relationship with a linear portion centered around one vaginal infectious dose_50_, the dose that infects 50% of the control animals (Figure 1). The results are well fit with the Michaelis-Menton equation: fraction infected = 1/(1 + (dose)^-1^) where dose is the viral dose expressed in units of ID_50_. This dose-response curve was similar to previous dose response curves obtained with other viral stocks, including the controls in our previous study with this model, which included a total of 550 mice [12] and lay along a nearly identical curve defined by the Michaelis-Menton equation (data not shown).

The close agreement with the Michaelis-Menton equation was consistent with "single-hit kinetics", i.e., infections being initiated by single virions. In addition, this close agreement suggests there was low variability between mice and low variability in the inoculum procedures since both these potential sources of variability would decrease the slope of the dose-response relationship.

### Sample size calculations

Statistical power calculations based on the dose-response relationship in Figure 1 indicated that with a viral dose of 1 ID_50 _in the control animals, 500, 210, and 100 animals divided equally between test and control animals are required to provide 80% power with alpha = 0.05, to detect an increase in susceptibility of 2-fold, 3-fold and 5-fold, respectively.

### Osmolality of test agents

The osmolality values reported in Table 1 are the means of three determinations. The average coefficient of variation of replicate determinations was 2.5%. The agents studied in the HEC placebo gel were only slightly hyperosmotic (~350 mOsm/kg compared with ~290 mOsm/kg for blood and most body fluids), with the exception of the formulations containing the humectant/solvent agents, glycerin and propylene glycol, which were formulated at 10% concentration and were moderately hyperosmotic (~1700 mOsm/kg). In contrast, 30% glycerin, KYWJ alone and KYWJ with 5% GML were extremely hyperosmotic, as were the undiluted main ingredients of KYWJ, propylene glycol and PEG-8.

**Table 1:** Summary of Susceptibility Studies: Mice challenged with 1 ID_50 _after pre-treatment with test agent or control

Test Agent administered 12 h before viral challenge	Fraction infected after Test Agent	Fraction infected after PBS Control	P value	Fold-increase in susceptibility	Osmolality (mOsm/kg)
Methylparaben/propylparaben (0.18%/0.02%) in HEC gel	19/60 (32%)	20/60 (33%)	1		374

Benzyl alcohol (1%) in HEC gel	26/60 (43%)	26/60 (43%)	1		338

Disodium EDTA (0.1%) in HEC gel	59/90 (66%)	52/90 (58%)	0.095	2*	376

Disodium EDTA (0.0186%) in HEC gel	10/60 (17%)	14/60 (23%)	0.49		341

Propylene glycol (10%) in HEC gel	38/60 (63%)	33/60 (55%)	0.46		1,770

Glycerin (10%) in HEC gel	15/60 (25%)	23/60 (38%)	0.17	2*	1,700

Glycerin (30%) in HEC gel	32/59 (54%)	20/59 (34%)	0.04	3	4,280

GML (5%) in K-Y Warming Jelly	59/60 (98%)	28/60 (47%)♦	< 0.0001	> 10	10,100
		
K-Y Warming Jelly (neat)	57/60 (95%)		< 0.0001	> 9	10,300

GML (5%) colloidal suspension in PBS	48/60 (80%)	33/60 (55%)	0.006	5	329

Propylene glycol (neat)	55/60 (92%)	28/59 (47%)♦	< 0.0001	10	9,990
		
Polyethylene glycol (PEG-8) (neat)	55/60 (92%)		< 0.0001	10	5,610

♦ Control for both agents in the adjacent column in a three-arm experiment.

* Fold-increases were calculated for these formulations because of their strong, though not statistically significant, trends toward increased susceptibility.

### Alterations in susceptibility to HSV-2 challenge

Table 1 shows the results of the susceptibility tests. The parabens and benzyl alcohol preservatives showed no increase in susceptibility. Animals treated with disodium EDTA at 0.1% showed a trend toward increased susceptibility that did not reach statistical significance (P = 0.095), and those treated with 0.0186% disodium EDTA showed no increase. A relatively low concentration (10%) of the humectant/solvent compound propylene glycol did not show an increase in susceptibility, but another humectant/solvent at the same concentration, 10% glycerin, showed a trend toward increased susceptibility, though it did not reach statistical significance.

In contrast, the last six agents shown in Table 1 all caused large and statistically significant increases in susceptibility. Five percent (5%) GML in KYWJ, and 5% GML in a colloidal suspension in PBS significantly increased susceptibility. The KYWJ vehicle and both its component humectants/solvents, propylene glycol (neat) and PEG-8 (neat), also caused large and highly significant increases in susceptibility. An intermediate concentration of another humectant/solvent, 30% glycerin in HEC also caused a significant increase in susceptibility.

Since 5% GML in KYWJ, and KYWJ alone both caused nearly all of the treated animals (more than 90%) to become infected, additional tests were performed with a lower-dose inoculum (0.1 ID_50_) to obtain a more accurate measure of the magnitudes of the susceptibility increases. This is the procedure used in our earlier studies of detergents and surface-active agents that also caused large increases in susceptibility [12]. The results are shown in Table 2. The treated animals were challenged with a ten-fold lower dose of virus than the control animals, 0.1 ID_50 _vs. 1 ID_50_, yet close to 50% of animals were infected in all three groups. Thus, the test agents increased susceptibility about 10-fold as indicated by the black square and the dashed arrow in Figure 1.

**Table 2:** Animals challenged (0.1 or 1.0 ID_50_) and infected after pre-treatment with test agent or PBS

Agent administered 12 h before viral challenge	Challenge dose 0.1 ID_50_	Challenge dose 1.0 ID_50_	Fold-increase susceptibility
GML in K-Y Warming Jelly	21/40 (53%)	*	10

K-Y Warming Jelly	18/39 (46%)	*	7

PBS	*	21/40 (53%)	Reference

*The exposures at challenge doses in these cells were not performed concurrently with the experiment reported in this Table, but results from previously performed experiments at these doses are shown in Table 1 and Fig. 1.

## Discussion

### Excipients are often *not *inactive

Excipients are thought of as "inactive ingredients" but often without evidence that they are inactive. Although excipients can serve important functions ancillary to active pharmaceutical ingredients (APIs) in topical microbicide products, they bear their own potential toxicities. We studied a broad range of excipient classes, namely, preservative, antioxidant, chelating, humectant, solublizing, and emulsifying excipients commonly used in vaginal [21] and other topical products in an assay that determines their impact on susceptibility to infection with HSV-2. We tested excipients in vehicles (i.e., HEC placebo gel; PBS solution) known to be free of toxicities that increased susceptibility [12], and also undiluted (neat) excipients, thus in isolation from any API, thereby preventing antiviral activity by the API from masking excipient-induced increases in susceptibility. Although it could be argued that an effective API would make excipient-induced increases in susceptibility irrelevant, in practice imperfect microbicide adherence, the unpredictable timing of intercourse, and the prolonged duration of altered susceptibility [12] are such that it cannot be assumed that effective levels of an API will be present throughout the period of increased susceptibility. Moreover, the API may not protect against all pathogens to which it or its excipients increase susceptibility.

Preservatives might be expected to be of particular concern since these agents kill or inhibit a broad range of microorganisms and may also have similar cellular toxicity. However, neither parabens nor benzyl alcohol caused a detectable increase in susceptibility in our model, despite an apparent association of benzyl alcohol with irritative symptoms in a Phase I study of cellulose sulfate [24]. Sorbic acid had previously been tested in our initial studies with this model [12] without detectable increase in susceptibility. In the present studies, we used the sorbic acid-preserved HEC placebo gel [16,17] as a vehicle for tests of other excipients, and thereby again confirmed the lack of increased susceptibility with sorbic acid and the HEC placebo gel vehicle when compared repeatedly to a PBS control. These findings also match the benign clinical safety profile and lack of effect on HIV transmission of the HEC placebo in an HIV efficacy trial, where it was directly compared to a no-gel arm [18].

EDTA is a chelating agent used as a preservative synergist at 0.01-0.1% and as an antioxidant synergist at 0.005 to 0.1% [21,25]. We studied disodium EDTA at two concentrations, 0.0186% and 0.1%. At 0.0186% (~ 0.5 mM) disodium EDTA showed no effect on susceptibility to HSV-2. However, at the higher end of the typically used concentration range (0.1%), there was a trend toward increased susceptibility to HSV-2 challenge (P = 0.095). Since EDTA has long been used to detach mucosal epithelia *in vitro *[26], the trend toward harm at the upper end of the typically used EDTA concentrations, though not statistically significant, raises concern about the safety of disodium EDTA at or beyond 0.1%.

Low molecular weight highly soluble organic compounds are commonly used as "humectants" in dermatological formulations and sexual lubricants to reduce evaporation-induced coldness and premature drying when aqueous formulations are spread over the skin, as well as for their lubricant character. The commonly used humectants glycerin, propylene glycol, and PEG-8 are also employed for their solvent and/or preservative-enhancing characters [25]. The slug mucosal irritation assay reported toxicity (irritation and damage) with high osmolality preparations, including Astroglide^® ^(containing glycerin and propylene glycol, osmolality ~5800 mOsm/kg), and an HEC gel with glycerin added at both 20 and 40% and an osmolality of 2200-4400 mOsm/kg) [11]. Several clinical studies likewise reported that other strongly hyperosmotic formulations disrupt the columnar epithelium of the rectum [27-29]. In our studies, the moderately hyperosmotic formulations of 10% glycerin and 10% propylene glycol did not show a significant increase in susceptibility, though 10% glycerin showed a trend toward, and 30% glycerin caused a significant increase in susceptibility. Moreover, consistent with the reports of toxicity with the extremely hyperosmotic formulations cited, we found that an extremely hyperosmotic vehicle, KYWJ (osmolality > 10,000 mOsm/kg) caused a 7-fold increase in susceptibility to HSV-2 (Table 2). Moreover, each of the primary constituents of this formulation, the humectant/solvents propylene glycol and PEG-8, greatly increased susceptibility. Both are markedly hyperosmotic, in the range of the complete KYWJ formulation, and their osmolality may mediate the toxicity. However, both are not only humectants, but also solvents, and their toxicity may additionally or alternatively be due to solvent properties or other characteristics. Indeed, propylene glycol has been demonstrated to have *in vitro *cellular toxicity at relatively low concentrations, independent of osmotic effects [30].

### GML and the hyperosmotic K-Y Warming Gel vehicle

Since our previous work with this model demonstrated very large increases in susceptibility after exposure to a diverse range of surfactants [12], and to the non-surfactant but membrane-active chlorhexidine [12], we tested the excipient glycerol monolaurate (GML). GML has surfactant, emulsifier, membrane-active, and penetration-enhancing actions. Moreover, GML is currently being studied as a novel microbicide API [19], hypothesized to act by down-regulating the activation, recruitment, and accumulation of HIV target cells [19]. GML is poorly soluble in water, and GML was formulated in the cited and present studies using the non-aqueous lubricant KYWJ as a vehicle, composed primarily of the low molecular weight humectant/solvents propylene glycol and PEG-8.

Using an inoculum of 1 ID_50_, we found that 5% GML formulated in KYWJ, caused a 10-fold increase in susceptibility to HSV-2, somewhat greater than the increase also observed after KYWJ vehicle alone. The rate of infection with 1 ID_50 _approached 100%, limiting an accurate estimation of the magnitude of susceptibility increase. Therefore we did additional experiments with a lower inoculum (0.1 ID_50_) and the results indicated the susceptibility increased 10-fold with GML in KYWJ and 7-fold with KYWJ alone. In light of the toxicity associated with KYWJ, we tested 5% GML without this vehicle, prepared as a colloidal suspension in PBS. Although GML formulated in PBS was nearly isotonic, it too significantly increased susceptibility (P < 0.006), indicating that both KYWJ and GML individually caused susceptibility-increasing toxicity.

The susceptibility-increasing toxicity of GML may be due to its surface-active and membrane-active properties. Indeed its effects on toxin production and signaling in bacteria and immune cells have been postulated to be due to intercalation of GML into cell membranes [31,32]. In light of the increase in susceptibility caused by a wide diversity of surfactant types in our prior study [12], and the surfactant nonoxynol-9 in a similar model [13], the increased susceptibility observed after exposure to GML is perhaps not unexpected. Yet it is notable that neither colposcopic nor histological abnormalities were detected after 6 months of daily vaginal administration of 5% GML in KYWJ in rhesus macaques [20]. However, in our prior studies of the surfactant nonoxynol-9, colposcopy was *normal *at the time of maximally increased susceptibility to HSV-2, twelve hours after exposure [12]. Moreover, our studies evaluated surfactant contact with columnar-like epithelium of the medroxyprogesterone acetate-treated mouse vagina since microbicides will contact human columnar epithelium in the endocervix [33]. Exposure to columnar epithelium will also occur on the face of the cervix when cervical ectopy is present. Examination of columnar epithelium (endocervix or ectopy) was not reported after chronic GML exposure in the GML toxicity study in macaques [20].

The present findings are examples of unexpected actions of excipients, and show that excipients are not necessarily inactive, nor non-toxic in mucosal contact. The previous demonstration of the effect of GML on signaling and toxin production in bacteria [32], its effect on cell immune cell proliferation [31], its activity as a penetration enhancer, the recent demonstration of its activity in inhibiting SIV [19], and the present demonstration of its action increasing susceptibility to genital herpes are examples of substantial activities of an excipient. Likewise the HSV-2 susceptibility-increasing effects of KYWJ, and the prior toxicity documented with another hypertonic lubricant, ID Glide^® ^[27], also illustrate the potential toxicity of vehicles (lubricant gels) even in the absence of an API.

We tested an intermediate concentration (30%) of the humectant/solvent glycerin because glycerin was toxic in this concentration range in an in vitro model [11], and is present in the gel formulation used in CAPRISA 004, where 1% tenofovir vaginal gel was reported to provide protection against both HIV and HSV-2. Thirty percent glycerin caused a significant increase in HSV-2 susceptibility in our model.

### Choice of challenge dose and estimation of fold-increase in susceptibility

In our previous publication [12] the surfactants tested caused very large increases in susceptibility, and we therefore used a viral dose of 0.1 ID_50 _to obtain a more accurate evaluation of the magnitudes of increased susceptibility. With agents of unknown, and perhaps only modest effects on susceptibility, an inoculum size of 1 ID_50 _is optimal for providing maximum statistical power to detect significant alterations in susceptibility. It is notable that a large number of animals was required to detect even fairly substantial increases in HSV-2 susceptibility (see Sample size calculations in Results). It is also worth emphasizing, as illustrated in Figure 1, it is inappropriate to calculate the magnitude of the increase in susceptibility simply by stating the ratio of the fractions of animals infected in the test and control groups. For example, with a challenge dose of 1 ID_50, _even an agent that increases susceptibility by 1000-fold could only double the fraction of animals infected. In contrast, human transmission of HSV-2 infections typically occurs with low probability of infection per coital event, implying 'low-dose' viral challenges. In such cases, increased susceptibility as defined here implies the probability of transmission per coital event will likely increase by the magnitude that the agent increases susceptibility; a 10-fold increase in susceptibility would lead to a 10-fold increase in human transmission rate.

### Limitations and strengths of this model

Our model has several limitations. Observations were made in mice rather than humans or non-human primates. Medroxyprogesterone acetate-treated mice have a thinned epithelium with living cells on the surface, mimicking columnar epithelium and probably with greater sensitivity to chemical damage or irritation than the multilayer squamous epithelium of the human vaginal epithelium. However, vaginal microbicides contact the columnar epithelium in the endocervix [33] via the well-documented mechanism of uterine peristaltic uptake [34-36] and also contact columnar epithelium on the ectocervix when cervical ectopy is present. It is appropriate for a screening test to be highly sensitive, and to mimic the exposures that are potentially most damaging. Moreover, vaginal microbicides will very likely be used to attempt rectal protection, where they will also contact columnar epithelium. For both these reasons, a model exposing a thin epithelium with living surface cells is highly relevant.

The challenge virus in this model is HSV-2, whereas HIV is the primary focus of most microbicide development efforts. However, it is plausible that toxicities that result in heightened susceptibility to HSV-2 may also increase susceptibility to HIV. Indeed results from our model with nonoxynol-9 and C31G, and a similar model [13] with nonoxynol-9, and cellulose sulfate, have correlated well with significantly harmful (nonoxynol-9, cellulose sulfate) or borderline harmful (C31G) clinical trial results. Moreover, HSV-2 is an important pathogen in its own right, and as a cofactor that substantially increases the risk of HIV acquisition. We therefore believe that a microbicide whose API, component excipients, or complete formulation significantly increase susceptibility to HSV-2 in mice cautions against advancing to clinical trials in humans.

In the present study, we investigated only a single time interval between application of the test agent and viral challenge. We elected to use the interval found to be associated with the maximum increase in sensitivity after exposure to the surfactant nonoxynol-9. However, we acknowledge that the timing of maximum susceptibility may vary depending on the agent tested.

Our model employs only a single exposure rather than repeated exposures over time. We chose this exposure protocol because in pilot experiments, multiple exposures with the surfactant microbicide nonoxynol-9 did not result in greater susceptibility than a single exposure (data not shown). In addition, the single-exposure model allows resources to be directed toward increasing the number of animals in groups, possibly providing greater overall sensitivity of the model. However, some agents may only show toxicities after multiple exposures, for example agents that are sensitizers, where inflammatory/immune-mediated toxicities may only be observed after sub-chronic or chronic dosing schedules.

Finally, neither this nor any model can be certain to detect all possible toxicities, and hence lack of toxicity in this model does not guarantee safety in human use. Conversely, when this or similar models detect increases in susceptibility to important STI pathogens, we believe such results should be considered important red flags cautioning against the use of an API, excipient, or completed formulation in the vagina or rectum.

Our model has the following strengths: It directly detects toxicities that result in heightened susceptibility to HSV-2 acquisition, rather than surrogate endpoints that are only *postulated *to cause increased susceptibility. It thus directly assesses what may be reasonably considered one of the most harmful potential toxicities of a candidate microbicide, a paradoxical *increase *in susceptibility to a serious sexually transmitted viral pathogen. Importantly, the results in this and similar models [13] correlate well with trends toward or statistically significant adverse effects of microbicides on HIV endpoints in clinical studies [1-4]. The model is efficient and sensitive, employing genital tract tissue with highly susceptible living surface cells and an optimized inoculum strength. It can use sufficient animals to provide statistical power to detect relatively small changes in susceptibility, increases in susceptibility increases as low as 3-5 fold over controls. We believe it can provide useful guidance on the suitability of APIs and excipients for use in vaginal microbicide formulations.

## Conclusions

Increased susceptibility caused by a candidate microbicide or excipient in this and similar animal models cautions against its advancement to clinical trials. Our results specifically caution against the use of KYWJ as a vehicle, glycerin at or above 30%, or high concentrations of PEG-8 or propylene glycol as excipients, and GML as an excipient or API. Although not significant, the trends toward harm with 0.1% disodium EDTA and 10% glycerin also argues for caution at or above these concentrations in microbicide or other vaginal formulations. Conversely, our results are reassuring regarding the suitability of the lower dose disodium EDTA and the other preservatives and humectants at the concentrations tested.

## Competing interests

TRM is an employee of ReProtect, Inc., the developer of BufferGel^®^, a spermicidal contraceptive, formerly but no longer being developed as a vaginal microbicide. TRM and RAC own equity in ReProtect. RJM, TEH, and MS have no competing interests.

## Authors' contributions

TRM and RAC initiated the study and participated in the design and analysis throughout. TRM drafted the manuscript. TH helped develop the procedures used in the mouse HSV-2 susceptibility model, and performed most of the experiments using this model. RJM participated in the design of the study, and the choice and formulation of the excipients studied. MS prepared and characterized the formulations studied. All authors read, helped revise, and approved the final manuscript.

## Pre-publication history

The pre-publication history for this paper can be accessed here:

http://www.biomedcentral.com/1471-2334/10/331/prepub
